# Pain Reduction in Linzagolix‐Treated Patients With Uterine Fibroids: A Secondary Mediation Analysis of the PRIMROSE 1 and 2 Phase 3 Trials

**DOI:** 10.1111/1471-0528.18190

**Published:** 2025-05-06

**Authors:** Sven Becker, Marie‐Madeleine Dolmans, Francisco Carmona Herrera, Felice Petraglia, Stefan P. Renner, Raluca Ionescu‐Ittu, Julien St‐Pierre, Mitra Boolell, Elke Bestel, Satoshi Hori, Jacques Donnez

**Affiliations:** ^1^ Department of Gynecology and Obstetrics University Hospital Frankfurt Frankfurt Germany; ^2^ Gynecology Research Laboratory, Institut de Recherche Expérimentale et Clinique, Department of Gynecology Université Catholique de Louvain Brussels Belgium; ^3^ Gynaecology Department, Clinic Institute of Gynaecology, Obstetrics and Neonatology (ICGON), Hospital Clinic of Barcelona University of Barcelona Barcelona Spain; ^4^ Obstetrics and Gynecology Unit, Department of Clinical Experimental and Biomedical Sciences University of Florence Florence Italy; ^5^ Department of Gynecology and Obstetrics Friedrich‐Alexander University Erlangen‐Nürnberg Erlangen Germany; ^6^ Department of Gynecology and Obstetrics Boeblingen Clinic, Hospital Sindelfingen‐Böblingen Böblingen Germany; ^7^ STATLOG Montreal Canada; ^8^ Department of Medical Affairs Theramex HQ Ltd London UK; ^9^ Société de Recherche Pour L'infertilité Université Catholique de Louvain Brussels Belgium

**Keywords:** fibroid volume, GnRH antagonist, heavy menstrual bleeding, linzagolix, mediation analysis, pain, uterine fibroids

## Abstract

**Objective:**

Among women with uterine fibroids (UFs), we assess the extent to which the linzagolix effect on pain alleviation is explained by its effect on reducing heavy menstrual bleeding (HMB) and fibroid volume (FV).

**Design:**

Post hoc analysis on the pooled data from two randomised double‐blind placebo‐controlled phase 3 trials.

**Setting:**

94 sites in the US (PRIMROSE 1 trial) and 95 sites in Europe/US (PRIMROSE 2 trial).

**Population:**

Women aged ≥ 18 years with ultrasound‐confirmed UFs and HMB (*n* = 1012).

**Methods:**

Participants were randomised to linzagolix (100 mg and 200 mg, with and without hormonal add‐back therapy) versus placebo. A post hoc mediation analysis was conducted on the pooled PRIMROSE 1 and PRIMROSE 2 data. The effect of linzagolix versus placebo on pain reduction was divided into three components (effect explained by HMB reduction associated with linzagolix, FV reduction associated with linzagolix, and remaining [not yet explained] treatment effect), with proportions of each component reported.

**Main Outcome Measures:**

The mediation analysis outcome was clinically significant pain reduction, defined as a change of ≥ 2 pain categories from baseline to Week 24 using the Numeric Rating Scale (pain categories: no pain (0), and mild (1–3), moderate (4–6), severe pain (7–10)).

**Results:**

In the mediation analysis, 28%–51% (depending on treatment arm) of linzagolix effect on pain reduction was explained by its effect on HMB reduction, while 2%–8% was explained by its effect on FV reduction. The residual proportion ranged between 44% and 67%, depending on treatment arm, and was statistically significant only in the linzagolix 200 mg without add‐back therapy arm (*p* = 0.002).

**Conclusions:**

This analysis showed that reductions in pain were significantly mediated by reductions in HMB (all doses) and FV (200 mg alone) in linzagolix‐treated women with UFs. Further research is needed to identify additional mediating factors.

**Trial Registration:**

ClinicalTrials.gov: NCT03070899 and NCT03070951

## 1 Introduction

Uterine fibroids (UFs) are common benign tumours that affect more than two thirds of women of reproductive age [1, 2]. Symptomatic UFs, which account for 25%–50% of cases [3], impose an estimated annual economic impact of $5.9–34.4 billion USD due to healthcare costs and productivity losses [4] along with a heavy clinical burden on the patient. Fibroid‐related pain, which, along with heavy menstrual bleeding (HMB) [5, 6, 7, 8, 9], is one of the most debilitating symptoms for affected women, remains an overlooked outcome in clinical research [10, 11, 12, 13].

Orally administered gonadotropin‐releasing hormone receptor (GnRH) antagonists are a new class of medications for UFs that were shown in clinical trials to significantly alleviate pain associated with UFs [14, 15, 16] and endometriosis [17], while also significantly reducing HMB and fibroid volume (FV) among women with UFs [18]. As GnRH antagonists do not have a direct analgesic effect, their impact on fibroid‐related pain is thought to occur through alleviation of associated symptoms such as HMB and FV reduction. HMB, the most common symptom of patients with UF, has been postulated to occur due to several mechanisms including an increased uterine surface area, increased vascularity and blood flow into the uterus, irregular myometrial contractility, endometrial ulceration, uterine venous ectasia caused by pressure from the fibroid, platelet dysfunction, elevated levels of matrix metalloproteinases in fibroids, alterations in the expression of angiogenic factors, as well as an increase in the secretion of TGF‐β3 [19, 20]. Furthermore, alterations in blood plasma levels of circulating interleukin (IL)‐13, IL‐17, and IL‐10 have been reported [21], which in turn could affect the immune function and inflammation implicated in the endometrial breakdown and repair. In summary, UFs are postulated to cause HMB and pain by the interference of myometrial contractility and vascularity due to the mechanical pressure caused by the fibroids themselves, or through alterations in the local paracrine signalling, haemostatic regulation and inflammatory changes of the endometrium overlying the leiomyoma [22]. However, the exact underlying pathways and interactions between UFs and pain are complex and poorly understood.

For the purposes of the current study, we hypothesised that the effect of GnRH antagonists on fibroid‐related pain is fully mediated by the effect of GnRH antagonists on reducing HMB and FV. To test this hypothesis and to advance current knowledge on mechanisms of fibroid‐related pain and fibroid‐related pain alleviation through treatment, we leveraged pooled data from two large, randomised placebo‐controlled Phase 3 trials, PRIMROSE 1 and PRIMROSE 2, that assessed the effect of linzagolix, a GnRH antagonist, on HMB (primary outcome) and FV and fibroid‐related pain (secondary outcomes) and conducted a mediation analysis.

## 2 Methods

### 2.1 Study Objective

The objective of the current study was to assess the extent to which linzagolix's effect on pain reduction at 24 weeks in the PRIMROSE 1 and PRIMROSE 2 trials could be explained by its effect on reducing HMB and FV.

### 2.2 Study Design and Participants

PRIMROSE 1 and PRIMROSE 2 were two similar, randomised, parallel, double‐blind, placebo‐controlled, Phase 3 trials (results of which were previously reported) [16]. For PRIMROSE 1, 574 women were recruited through 94 sites (hospitals, clinics, and private research facilities) in the United States (US) from May 2017 to October 2020; for PRIMROSE 2, 535 women were recruited through 95 sites in Europe and the US from June 2017 to May 2020. Women were eligible for inclusion in PRIMROSE 1 and PRIMROSE 2 if they were aged ≥ 18 years; had ultrasound‐confirmed fibroids; had at least one fibroid of ≥ 2 cm diameter or multiple small fibroids with a calculated uterus volume > 200 cm^3^; had no fibroid with a diameter > 12 cm; and had HMB for ≥ 2 cycles. After inclusion and exclusion criteria, a total of 511 and 501 women from PRIMROSE 1 and PRIMROSE 2 respectively were randomised 1:1:1:1:1 to five treatment arms: (a) placebo, (b) 100 mg linzagolix without add‐back therapy (ABT), (c) 100 mg linzagolix with ABT, (d) 200 mg linzagolix without ABT, (e) 200 mg linzagolix with ABT. The ABT consisted of 1 mg estradiol and 0.5 mg norethisterone acetate taken once a day. The primary efficacy endpoint of the PRIMROSE 1 and 2 trials was menstrual blood loss of 80 mL or less and a 50% or more reduction in menstrual blood loss from baseline in the 28 days before Week 24. Key secondary endpoints at Week 24 were the time to reduced menstrual blood loss, incidence of amenorrhoea, time to amenorrhoea, the number of days of uterine bleeding in the last 28 days before Week 24, and haemoglobin concentrations in participants who were anaemic at baseline.

The current post hoc analysis focused on changes in outcomes from baseline to Week 24 in the pooled samples of participants in PRIMROSE 1 and PRIMROSE 2 trials who received ≥ 1 dose of the randomised treatment (analysis set; *n* = 1012). All participants provided written informed consent, and the original PRIMROSE 1 and 2 protocols were approved by a central review board (Quorum Review, Seattle, WA, USA) and an Ethics Committee or Institutional Review Board at each participating centre. The trials were conducted in accordance with guidelines of the International Council for Harmonisation and the principles of the Declaration of Helsinki.

### 2.3 Outcomes and Measurements

In this post hoc mediation analysis, clinically significant pain reduction from baseline to week 24 was the outcome of interest, while clinically significant HMB reduction and FV reduction were assessed as intermediary outcomes that explained the effect of linzagolix on pain reduction. All three outcomes (pain, MBL, FV) were measured in accordance with the protocol definitions in the PRIMROSE 1 and PRIMROSE 2 trials, as described below. The main results of the PRIMROSE 1 and PRIMROSE 2 trials, with MBL as the primary outcome and pain/FV as secondary outcomes, are reported elsewhere [16].

Self‐reported fibroid‐related pain was assessed at Day 1, and Weeks 12 and 24 with the prompt, “please rate your pain related to your uterine fibroids during the last 4 weeks”. Participants scored pain on a Numeric Rating Scale (NRS) from 0 (no pain) to 10 (worst possible pain) and pain categories were subsequently grouped into four levels (none: 0, mild: 1–3, moderate: 4–6, and severe: 7–10). Pain reduction was defined as a dichotomous variable (yes/no), with ‘yes’ corresponding to clinically significant change in pain at 24 weeks from baseline, defined as a decrease in pain by ≥ 2 pain categories. Subjects with baseline pain ≤ 3 (none or mild pain) were included in the main analysis to preserve randomisation, despite their inability to experience clinically significant pain reduction. A sensitivity analysis was also conducted among participants with only moderate or severe pain at baseline.

Menstrual blood loss was measured by the alkaline haematin method in a central laboratory masked to the study treatment. HMB reduction was defined as a dichotomous variable (yes/no), with “yes” corresponding to clinically significant change in menstrual blood loss at 24 weeks from baseline, defined as both (a) ≥ 50% reduction in menstrual blood loss from baseline at 24 weeks, and (b) menstrual blood loss ≤ 80 mL at Week 24).

Fibroid dimensions were estimated using ultrasonography, and volumes were calculated by the prolate ellipsoid formula: length×height×width×0.523. At each visit, the three largest fibroids (not necessarily the same from previous visits) were included in the total FV calculation. Total FV reduction thus calculated was log‐transformed due to their highly skewed distribution. Given the log transformation, FV reduction from baseline was quantified as 100% minus the ratio of current volume (Weeks 12 and 24) to baseline volume, which can be interpreted as the percentage of volume reduction from baseline.

### 2.4 Statistical Analyses

A mediation analysis [23, 24] was conducted on the pooled PRIMROSE 1 and PRIMROSE 2 data to assess whether pain reduction from the linzagolix effect were mediated by reductions in FV and HMB. The mediation analysis methodology dissects the total effect of treatment on pain reduction into three distinct components:

- The effect of treatment on pain reduction is explained by HMB reduction.
- The effect of treatment on pain reduction is explained by FV reduction.
- The residual effect of treatment on pain, not mediated by FV or HMB reduction, encompasses possible direct treatment effects and/or effects explained by other mediators.

**FIGURE 1:**
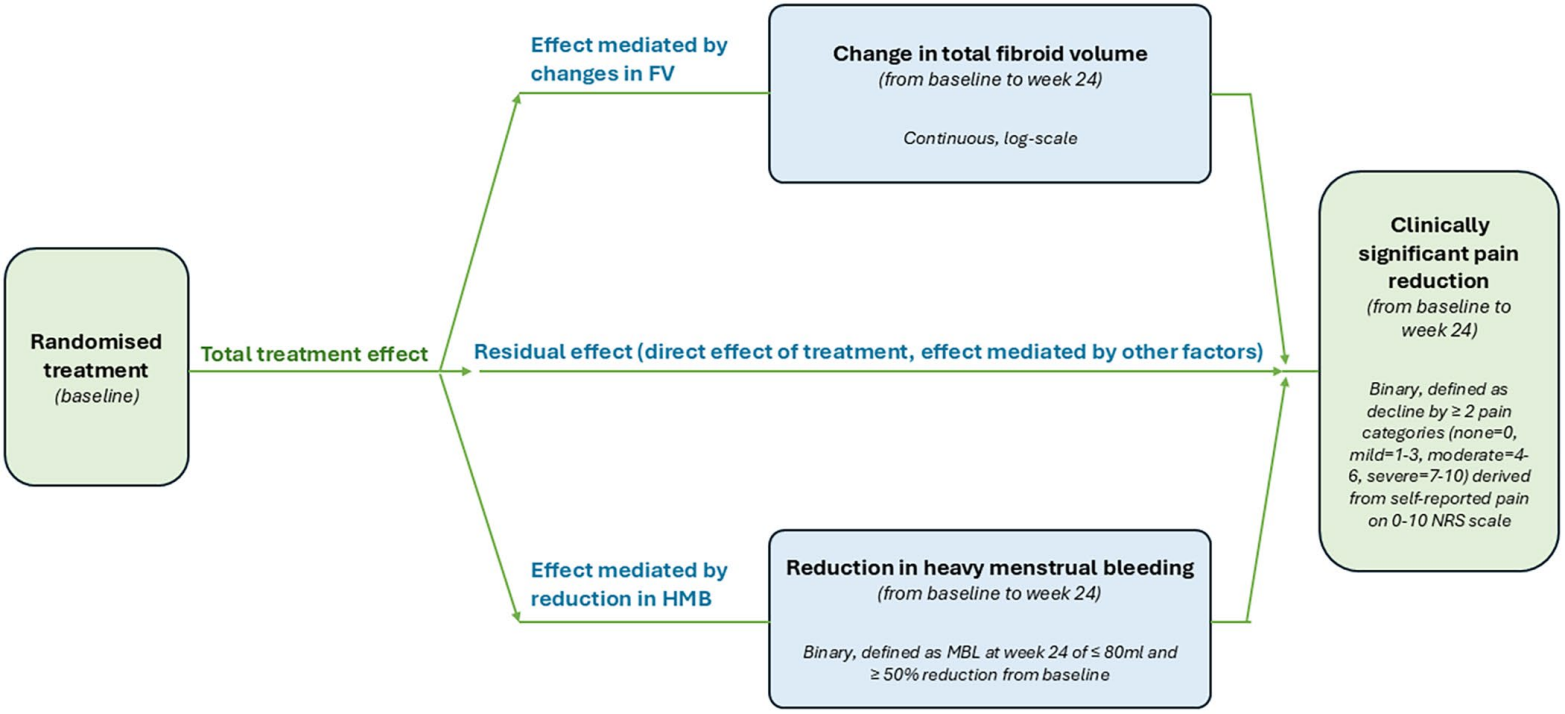
Hypothesized conceptual framework for the mediation analysis. FV, Fibroid volume; HMB, heavy menstrual bleeding; MBL, menstrual blood loss; NRS, numeric rating scale.

The mediation analysis (Figure 1) was conducted in three analytical steps. First, a logistic regression model was fitted to evaluate the overall impact of the treatment on clinically significant pain reduction (yes/no). Second, we assessed the treatment effect on pain, accounting for HMB and FV reductions by incorporating HMB and FV reductions as covariates in the logistic model. Lastly, we assessed the treatment effect on each mediator separately: HMB reduction was modelled as a binary outcome using logistic regression, while the decrease in FV was treated as a continuous outcome in a linear regression model, both in accordance with the original PRIMROSE study protocol. A separate sensitivity analysis was also conducted with menstrual blood loss as a continuous variable. All models were adjusted for race, age, BMI, baseline FV levels, baseline menstrual blood loss, analgesic use at baseline or in the 28 days leading to the 24 weeks visit, and PRIMROSE 1 versus PRIMROSE 2 study.

Results were reported as adjusted odds ratios (OR) with the associated 95% confidence intervals (CI). Individual active‐versus‐placebo effect comparisons were conducted at 24 weeks using a Bonferroni type 1 error of 0.0125 to account for the multiplicity of the four active treatment arms. Statistical analyses were performed using R (version 4.2.3, R Foundation for Statistical Computing, Vienna, Austria), and the *mma* package [25] was used to perform the mediation analyses.

## 3 Results

### 3.1 Patient Characteristics

Baseline characteristics of participants in the PRIMROSE 1 and PRIMROSE 2 trials have been reported previously for each trial separately [16]. Table 1 describes the baseline characteristics of participants in the full analysis set (*n* = 1012) with pooled data from PRIMROSE 1 (*n* = 511) and PRIMROSE 2 (*n* = 501) trials. Mean age was 42.3 years, 64% were White, mean BMI was 29.8 kg/m^2^, median total FV was 53 cm^3^, and 13% reported using analgesic medications for UFs at baseline. Most patients (91%) experienced some pain at baseline (abdominal pain: 68%; lower back pain: 50%; pain during intercourse: 28%), with 75% experiencing moderate‐to‐severe pain and 43% experiencing severe pain. Patient characteristics were well balanced at baseline for the randomised participants in the analysis set and for the subset of patients with only moderate or severe pain at baseline (*n* = 761; Table S1).

**TABLE 1:** Demographic and clinical characteristics of the PRIMROSE 1 and PRIMROSE 2 participants at the time of randomisation (baseline; *N* = 1012).

	Placebo (*N* = 205)	Linzagolix 100 mg (*N* = 191)	Linzagolix 100 mg + ABT (*N* = 208)	Linzagolix 200 mg (*N* = 207)	Linzagolix 200 mg + ABT (*N* = 201)	Total (*N* = 1012)
Study ID, *n* (%)						
PRIMROSE 1	103 (50%)	94 (49%)	107 (51%)	104 (50%)	103 (51%)	511 (50%)
PRIMROSE 2	102 (50%)	97 (51%)	101 (49%)	103 (50%)	98 (49%)	501 (50%)
Age, mean (SD)	42.5 (5.5)	42.4 (5.7)	42.1 (5.6)	42.0 (5.9)	42.4 (5.5)	42.3 (5.6)
Race, *n* (%)						
Black	70 (34%)	64 (34%)	75 (36%)	73 (35%)	67 (33%)	349 (34%)
White	134 (65%)	121 (63%)	127 (61%)	131 (63%)	130 (65%)	643 (64%)
BMI (kg/m^2^), mean (SD)	29.45 (6.76)	30.27 (7.18)	29.89 (6.75)	29.50 (6.60)	29.95 (7.13)	29.80 (6.87)
Total fibroid volume (mL)^a^, median (Q1 – Q3)	52 (23–129)	65 (26–141)	50 (21–135)	44 (19–117)	58 (24–121)	53 (22–127)
Pain NRS^b^, mean (SD)	5.33 (2.81)	5.79 (2.69)	5.73 (2.75)	5.98 (2.89)	5.43 (2.93)	5.65 (2.82)
Pain^c^, *n* (%)						
None	14 (7%)	11 (6%)	13 (6%)	11 (5%)	15 (7%)	64 (6%)
Mild	38 (19%)	25 (13%)	29 (14%)	31 (15%)	39 (19%)	162 (16%)
Moderate	74 (36%)	63 (33%)	73 (35%)	59 (29%)	58 (29%)	327 (32%)
Severe	77 (38%)	85 (45%)	89 (43%)	98 (47%)	85 (42%)	434 (43%)
Pain symptoms						
Abdominal pain	138 (67%)	130 (68%)	158 (76%)	137 (66%)	124 (62%)	687 (68%)
Lower back pain	94 (46%)	100 (52%)	112 (54%)	105 (51%)	97 (49%)	508 (50%)
Pain during intercourse	55 (27%)	45 (24%)	65 (31%)	59 (28%)	56 (28%)	280 (28%)
Menstrual blood loss (mL), median (Q1 – Q3)	172 (126–239)	179 (129–269)	161 (120–261)	158 (125–265)	164 (122–226)	164 (123–250)
Baseline use of analgesic medications, *n* (%)	21 (10%)	28 (15%)	29 (14%)	24 (12%)	27 (13%)	129 (13%)

Abbreviations: ABT, add‐back therapy; BMI, body mass index; NRS, numeric rating scale; SD, standard deviation.

^a^ Fibroid dimensions were estimated using ultrasonography and volumes were calculated by the prolate ellipsoid formula: length×height×width×0.523. Up to three largest fibroids were included in the total FV calculation. 1 mL = 1 cm^3^.

^b^ Pain numeric rating scale (NRS) was assessed by patient self‐reporting using an electronic diary with a numeric rating scale from 0 (no pain) to 10 (worst possible pain).

^c^ Pain NRS was categorised into none (0), mild (1–3), moderate (4–6) and severe (7–10).

### 3.2 Change in Outcomes From Baseline

In the full analysis set with pooled data from PRIMROSE 1 and PRIMROSE 2 trials, the proportions of women with clinically significant HMB reduction at 24 weeks were significantly higher in the four linzagolix treatment arms (range 56.5%–84.6%) compared to placebo (32.2%; Table 2 and Figure S1). HMB reduction rates were higher in linzagolix treatment arms with higher doses and those with ABT: 56.5% (95% CI 49.5%–63·6%) for linzagolix 100 mg without ABT, 71.6% (65.5%–77.8%) for linzagolix 100 mg with ABT, 74.4% (68.5%–80.3%) for linzagolix 200 mg without ABT, and 84.6% (79.6%–89.6%) for linzagolix 200 mg with ABT (Table 2 and Figure S1).

**TABLE 2:** Comparison of heavy menstrual bleeding, fibroid volume, and pain reduction from baseline to 24 weeks between treatment arms and placebo (*N* = 1012).

	Placebo (*N* = 205)	Linzagolix 100 mg (*N* = 191)	Linzagolix 100 mg + ABT (*N* = 208)	Linzagolix 200 mg (*N* = 207)	Linzagolix 200 mg + ABT (*N* = 201)
Patients with clinically significant HMB reduction					
*n* (%)	66 (32.2%)	108 (56.5%)	149 (71.6%)	154 (74.4%)	170 (84.6%)
95% CI	(25.8%; 38.6%)	(49.5%; 63.6%)	(65.5%; 77.8%)	(68.5%; 80.3%)	(79.6%; 89.6%)
*p*‐value^a^, ^b^	—	< 0.001*	< 0.001*	< 0.001*	< 0.001*
Mean FV reduction from baseline to week 24 (%)					
Patients with FV measurement at week 24, *N*	147	137	136	156	146
Mean	13.8%	32.0%	21.1%	52.4%	30.6%
(95% CI)	(−0.63%; 26.1%)	(22.4%; 40.4%)	(11.0%; 30.1%)	(43.9%; 59.6%)	(18.6%; 40.9%)
*p*‐value^a^, ^b^		0.026	0.33	< 0.001*	0.038
Patients with clinically significant pain reduction					
*n* (%)	14 (6.8%)	36 (18.8%)	44 (21.2%)	64 (30.9%)	50 (24.9%)
95% CI	(3.38%; 10.3%)	(13.3%; 24.4%)	(15.6%; 26.7%)	(24.6%; 37.2%)	(18.9%; 30.9%)
*p*‐value^a^, ^b^	—	< 0.001*	< 0.001*	< 0.001*	< 0.001*

Abbreviations: ABT, add‐back therapy; CI, confidence interval; FV, fibroid volume; HMB, heavy menstrual bleeding.

^a^ *p*‐value for treatment effect versus placebo using a logistic regression model for HMB and Pain responses, and a linear regression model for FV reduction.

^b^ All models were adjusted for race, age, BMI, baseline FV, baseline menstrual blood loss, use of analgesic therapy at baseline or in the 28 days leading to the 24 weeks visit, and PRIMROSE 1 versus PRIMROSE 2 study.

* Significant at the 0.0125 level.

The mean decrease in FV from baseline to Week 24 was higher in all four linzagolix treatment arms (range 21.1%–52.4%) compared to the placebo arm (13.8%; Table 2 and Figure S2), although the only statistically significant result at the 0.0125 level was for the 200 mg without ABT arm (mean FV reduction of 52.4%; 95% CI 43.9%–59.6%). In all treatment arms, the largest decline in mean FV occurred in the first 12 weeks after treatment start. The mean FV continued to decline between Weeks 12 and 24, but at a slower rate (Figure S2).

The proportions of women with clinically significant pain reduction at Week 24 were significantly larger in all four linzagolix treatment arms (range 18.8%–30.9%) compared to the placebo arm (6.8%; Table 2 and Figure S3). Pain reduction rates were larger for higher linzagolix doses, without a clear pattern for the addition of ABT: 18.8% (95% CI 13.3%–24.4%) for linzagolix 100 mg without ABT, 21.2% (15.6%–26.7%) for linzagolix 100 mg with ABT, 30.9% (24.6%–37.2%) for linzagolix 200 mg without ABT, and 24.9% (18.9%–30.9%) for linzagolix 200 mg with ABT (Table 2 and Figure S3). As expected, more participants experienced clinically significant pain reduction at Week 24 when the sample was restricted to patients with moderate and severe pain at baseline than in the main analyses with the full analysis set (Figure S4).

### 3.3 Mediation Analysis: Effect of Treatment on Pain Reduction Mediated by Reductions in HMB and FV

The mediation analysis assessed the total treatment effect on reduction of clinically significant pain mediated through changes in HMB, FV and other unknown factors (residual effect). A significant effect on pain reduction was observed in all four treatment arms compared to placebo, with ORs for the total effect ranging from 3.83 (for linzagolix 100 mg without ABT) to 8.37 (for linzagolix 200 mg without ABT) (Table 3). The HMB reduction associated with the treatment explained between 28% and 51% of the total effect of treatment on pain reduction depending on the treatment arm (with higher contributions for treatments that included higher linzagolix doses and ABT), while the decrease in FV associated with the treatment explained between 2% and 8% of the total effect (with higher contributions for treatment arms that included higher linzagolix doses and lower contributions for treatment arms that included ABT) (Table 3).

**TABLE 3:** Mediation analysis of linzagolix on pain reduction from baseline at 24 weeks with fibroid volume and heavy menstrual bleeding reductions as mediators (*N* = 1012).

Treatment effect on clinically significant pain reduction^a^	Odds ratio (95% CI)^b^, ^c^	*p*‐values	Percentage of total effects^d^
**Linzagolix 100 mg vs. placebo**			
Total effect of linzagolix 100 mg vs. placebo	3.83 (1.74–8.92)	0.001*	
Effect mediated by FV and HMB reductions	1.57 (1.24–2.08)	0.001*	33%
Effect mediated by FV reduction	1.08 (1.00–1.19)	0.038	6%
Effect mediated by HMB reduction	1.45 (1.18–1.88)	0.002*	28%
Residual treatment effect^e^	2.45 (1.14–5.72)	0.018	67%
**Linzagolix 100 mg + ABT vs. placebo**			
Total effect of linzagolix 100 mg + ABT vs. placebo	5.28 (2.55–12.70)	< 0.001*	
Effect mediated by FV and HMB reductions	2.25 (1.58–3.40)	< 0.001*	49%
Effect mediated by FV reduction	1.03 (0.96–1.11)	0.500	2%
Effect mediated by HMB reduction	2.20 (1.57–3.29)	< 0.001*	47%
Residual treatment effect^e^	2.34 (1.11–5.52)	0.020	51%
**Linzagolix 200 mg vs. placebo**			
Total effect of linzagolix 200 mg vs. placebo	8.37 (3.94–19.89)	< 0.001*	
Effect mediated by FV and HMB reductions	2.51 (1.80–3.89)	< 0.001*	43%
Effect mediated by FV reduction	1.19 (1.05–1.38)	0.002*	8%
Effect mediated by HMB reduction	2.11 (1.55–3.10)	< 0.001*	35%
Residual treatment effect^e^	3.34 (1.63–7.61)	0.002*	57%
**Linzagolix 200 mg + ABT vs. placebo**			
Total effect of linzagolix 200 mg + ABT vs. placebo	5.61 (2.65–12.90)	< 0.001*	
Effect mediated by FV and HMB reductions	2.62 (1.80–4.14)	< 0.001*	56%
Effect mediated by FV reduction	1.08 (1.00–1.19)	0.056	4%
Effect mediated by HMB reduction	2.43 (1.68–3.75)	< 0.001*	51%
Residual treatment effect^e^	2.15 (1.00–4.98)	0.048	44%

Abbreviations: ABT, add‐back therapy; CI, confidence interval; FV, fibroid volume; HMB, heavy menstrual bleeding.

^a^ For the purposes of the current study, clinically significant pain reduction was defined as a decline by ≥ 2 pain categories derived from self‐reported pain on a 0–10 scale (none = 0, mild = 1–3, moderate = 4–6, severe = 7–10).

^b^ Effects are additive in the log odds ratio scale (i.e., mediated effects + residual effects add up to total effect).

^c^ Interpretation of odds ratios (OR) > 1: the odds of experiencing a clinically significant reduction in pain are *x* higher (*x* = OR value) for patients in the treatment arm of interest compared to patients in the placebo arm.

^d^ Percentages of total effect are computed on the log odds ratio scale using the effect size estimates.

^e^ Residual treatment effects are a combination of the direct treatment effect on pain reduction and the treatment effect mediated by factors other than FV and HMB reductions.

* Significant at the 0.0125 level.

The treatment effects on pain reduction mediated by HMB reduction were statistically significant in all four linzagolix treatment arms, with OR (95% CI) ranging from 1.45 (1.18–1.88) for linzagolix 100 mg without ABT to 2.43 (1.68–3.75) for linzagolix 200 mg with ABT. The indirect treatment effect on pain reduction mediated by FV reduction was significant only for linzagolix 200 mg without ABT (OR 1.19, 1.05–1.38) (Table 3). In this treatment arm, the reported estimated OR of 1.19 signifies that the odds of experiencing a reduction in pain at Week 24 attributable to FV reduction are 19% higher in the linzagolix 200 mg arm compared to the placebo group. The percentage of the total treatment effect on pain reduction that was not associated with either a decrease in FV or HMB reduction (i.e., residual treatment effect) was estimated at 67%, 51%, 57%, and 44% for linzagolix 100 mg without ABT, linzagolix 100 mg with ABT, linzagolix 200 mg without ABT, and linzagolix 200 mg with ABT, respectively (Table 3). Statistical significance for the residual treatment effect was only observed in the linzagolix 200 mg without ABT arm (*p* = 0.002). A sensitivity analysis was also conducted using menstrual blood loss as a continuous variable, with findings similar to the main mediation analysis (Table S2).

A further sensitivity analysis conducted on the subset of participants with only moderate or severe pain at baseline showed similar findings to the mediation analysis of the full sample (Table S3).

## 4 Discussion

### 4.1 Main Findings

Pooled data from the PRIMROSE 1 and PRIMROSE 2 trials revealed that almost half (43%) of women with symptomatic UFs with HMB experienced severe pain at baseline. Reanalysis of the data was consistent with prior findings on linzagolix's efficacy in reducing pain, HMB, and FV (200 mg dose without ABT) after 24 weeks compared to placebo [16]. A novel mediation analysis also revealed that HMB and FV reductions were important mediators of fibroid‐related pain reduction observed among women treated with linzagolix as compared to placebo. Indeed, HMB reduction explained between 28% and 51% of the total effect of linzagolix versus placebo on pain reduction, depending on the treatment groups, while FV reduction explained 8% of the effect in the 200 mg without ABT dose group. Moreover, the odds of experiencing a reduction in pain at week 24 attributable to FV reduction were 19% higher in the linzagolix 200 mg arm compared to the placebo group. The unexplained effect of linzagolix on pain reduction, which was not due to HMB or FV reductions, varied from 44% to 67% across treatment arms, although statistical significance for the residual treatment effect was only seen in the linzagolix 200 mg without add‐back therapy (ABT) arm (*p* = 0.002).

### 4.2 Strengths and Limitations

The primary strength of this study lies in its contribution to the understanding of pain mechanisms in UFs, an often overlooked yet crucial outcome for the affected women [7, 9, 12, 26]. In addition to demonstrating for the first time that HMB and FV are important mediators of linzagolix's effect on fibroid‐related pain, the current study also highlights the ongoing gaps in our understanding, as evidenced by the relatively large residual effect on fibroid‐related pain that remained unexplained. This, however, opens new research avenues to identify other mechanisms and mediators through which linzagolix reduces fibroid‐related pain. Depending on the findings, such future research may provide insights that will help develop more effective strategies for managing fibroid‐related pain. Finally, the post hoc mediation analysis used in this study was possible by pooling data from PRIMROSE 1 and PRIMROSE 2 trials to increase the power for the mediation analysis.

The study is also subject to a few limitations. First, pooling of the data from the PRIMROSE 1 and PRIMROSE 2 studies may lead to bias if there are differences between participants in the two PRIMROSE trials that are not accounted for in the analysis. To mitigate this risk of bias, the current analyses were adjusted for key potential confounders, including race, age, BMI, baseline FV levels, baseline menstrual blood loss, analgesic use at baseline or in the 28 days leading to the 24 weeks visit, and PRIMROSE 1 versus PRIMROSE 2 study. Second, although analyses showed that HMB and FV reduction accounted for an important proportion of pain reduction, the remaining portion remains unknown, highlighting the need for further research to explore other possible mediators. Third, improvement in fibroid‐related pain was evaluated globally as perceived by the patient and not by symptom type or location (e.g., abdominal pain, lower back pain, pain on sexual intercourse) thus, making it unclear whether location or type of pain is a mediator of clinically significant pain reduction. Fourth, the current single‐level mediation model only allowed for the exploration of concomitant intermediate variables, meaning that more complex variables such as estradiol levels (which are likely to affect both HMB and FV) were not incorporated in the existing model. Lastly, although the study populations were randomised and representative of women with symptomatic UFs, the real‐world generalisability of the findings from this study is limited by the high proportion of ineligible patients; notably, women were excluded if they did not meet the strict criteria for HMB (≥ 80 mL of MBL per cycle for ≥ 2 cycles), if they had a uterus volume of > 200 cm^3^, or if they had fibroids of ≥ 12 cm.

### 4.3 Interpretation

Despite being often overlooked in clinical practice and clinical research [10, 11, 12], UF‐related pain is a bothersome symptom that significantly affects women's well‐being and quality of life [7, 11]. Indeed, nearly two‐thirds of women with UFs reported that their symptoms negatively impacted their lives in the past year, affecting their sexual life (42.9%), work performance (27.7%), and relationships & family (27.2%) [7], which has only been compounded further by the delayed treatment during the COVID‐19 pandemic [27]. In the PRIMROSE trials, almost half of women with fibroids and HMB reported severe pain, which aligns with other studies showing higher pain rates in women with UFs than those without UFs [7, 9, 28]. The prevalence and intensity of pain in women with symptomatic UFs underscore the need for a better understanding of pain mechanisms in UF, as well as a more standardised approach to research that allows for more meaningful comparisons to occur between studies [29]. The current study found that HMB reduction was a stronger mediator of the linzagolix treatment effect on pain compared to FV reduction, which is consistent with linzagolix's more pronounced impact on HMB reduction compared to FV reduction across all treatment groups. As cramps and pain during menstruation are common even in healthier populations [30, 31, 32], it is not surprising that reducing HMB would result in lessening of menstrual pain, particularly when bleeding is reduced to amenorrhoea. Other studies corroborate this, showing a link between prolonged bleeding, dysmenorrhoea, and increased pain risk [32]. Conversely, the relationship between FV and pain is inconsistent [9], suggesting a correlation only under certain conditions, such as with exceptionally large fibroids or specific fibroid locations.

The mediation analysis also found that a relatively large proportion of the total effect of linzagolix on pain reduction (between 44% and 67%, depending on the treatment group) remained unexplained after accounting for the effect mediated by HMB or FV reductions, with a statistically significant residual effect in the linzagolix 200 mg without ABT arm (*p* = 0.002). While other mechanisms through which linzagolix leads to pain reduction cannot be tested with the current single‐level mediation model and thus remain speculative, it is important to recognise that unexpected findings often serve as a catalyst for generating new hypotheses, allowing for the development and testing of new theories. For example, in the current study we do not have information on how many women included in PRIMROSE had comorbid endometriosis/adenomyosis, two conditions that have pain manifestations that overlap partially, but not completely, with the pain manifestations in UFs [33]. However, estimates from the literature indicate that between 12% and 86% of women with UFs may have comorbid endometriosis [33] and approximately half of women with UFs may have comorbid adenomyosis [34], suggesting some patients in the PRIMROSE trials may have had comorbid endometriosis/adenomyosis. If this is true and linzagolix has a therapeutic effect for pain in endometriosis as it has been shown in the EDELWEISS 3 trial [17], we can hypothesise that part of the unexpected effect of linzagolix on pain reduction in UFs could be explained by linzagolix's effect on endometriosis‐related pain reduction, although this was not formally explored in this study.

Further, methodological reasons may have also led to an underestimation of the relative contribution of HMB and FV reduction to the total effect of linzagolix on pain and thus the overestimation of the portion of the total effect that remained unexplained. First, changes in HMB and pain were analysed as binary variables (for consistency with the protocol for the main PRIMROSE analyses) which may have led to loss of information and misclassification. A sensitivity analysis using menstrual blood loss as a continuous variable, however, did not reveal any differences to the main mediation analysis findings. Second, FV was assessed using ultrasonography and relied on the three largest fibroids rather than all fibroids, which could have also led to loss of information and misclassification. Third, the subjective nature of self‐assessed pain possibly impacted the observed correlations between pain, fibroid, and HMB reductions. Finally, while other patient characteristics may influence linzagolix effects estimated in the mediation analysis (e.g., total treatment effect on pain is likely larger in subgroups with higher pain at baseline; the effect of linzagolix on pain reduction mediated by FV may be higher in women with large or very large fibroids) the current mediation analysis focused on the overall effects without accounting for possible effect modifiers.

## 5 Conclusion

This secondary analysis of the two PRIMROSE trials was conducted to assess whether observed pain reduction at 24 weeks in women treated with linzagolix versus placebo was potentially mediated by the effect of linzagolix on the decrease in FV and HMB reduction. Our results showed that substantial reductions in HMB and, to a lesser extent, FV attributable to linzagolix were correlated with the observed pain reduction among patients treated with linzagolix versus placebo. The remaining residual treatment effect on pain that was not attributable to the observed FV and HMB reductions suggests that additional factors that were not accounted for in this study may contribute to a potential effect on pain reduction. Further research is therefore needed in better understanding underlying clinical factors (e.g., myometrial distortion, irregular myometrial contraction or inflammation), as well as mechanistic pathways (e.g., alterations in matrix metalloproteinases, angiogenic factors and circulating interleukin levels) to better understand the pathophysiology of UF and their association with HMB and pain in women with symptomatic UF.

## Author Contributions

J.D. and E.B. contributed to the protocol and design of the PRIMROSE 1 and PRIMROSE 2 trials. E.B. contributed to the PRIMROSE 1 and PRIMROSE 2 data collection. S.H. and E.B. contributed to the review of the clinical trial data. M.B., E.B., S.H. contributed to the data acquisition for the purposes of the current post hoc analysis of the pooled PRIMROSE 1 and PRIMROSE 2 trial data. R.I.‐I., J.S.‐P., M.B., E.B., S.H. contributed to the conception, design, and planning of the current study. J.S.‐P. performed the analyses for the current study. All authors had substantive contributions to the interpretation of results. All authors critically reviewed the manuscript for important intellectual content. All authors have read and approved the final manuscript.

## Ethics Statement

This is a post hoc analysis of data from PRIMROSE 1 and PRIMROSE 2 clinical trials, whose protocols were approved by a central review board (Quorum Review, Seattle, WA, USA) and an Ethics Committee or Institutional Review Board at each participating centre.

## Consent

The PRIMROSE 1 and PRIMROSE 2 trials were conducted in accordance with guidelines of the International Council for Harmonisation and the principles of the Declaration of Helsinki, and all participants provided written informed consent.

## Conflicts of Interest

S.B. had received honoraria for lectures from Theramex. M.‐M.D. had received honoraria for lectures from Theramex and Gedeon Richter. F.C.H. had received honoraria for lectures from Theramex. F.P. had received honoraria for lectures from Theramex. S.P.R. had received honoraria for consultancy and lectures from Theramex and Gedeon Richter. J.S.‐P. and R.I.‐I. are employees of STATLOG, a consultancy company in the biomedical arena, that has received funds from Theramex for the conduct of the current study. M.B. is an employee of Theramex. E.B. is an employee of Theramex. S.H. is an employee of Theramex. J.D. had received honoraria for lectures and consultancy fees from Gedeon Richter, ObsEva, and Theramex.

## Supporting information


Appendix S1.
**Table S1.** Demographic and clinical characteristics of participants with only moderate or severe pain at baseline (*N* = 761). ABT, add‐back therapy; BMI, body mass index; NRS, numeric rating scale; SD, standard deviation ^1^Fibroid dimensions were estimated using ultrasonography; volumes were calculated by the prolate ellipsoid formula: length×height×width×0.523. Up to the three largest fibroids were included in the total FV calculation. 1 mL = 1 cm^3^. ^2^Pain numeric rating scale (NRS) was assessed by patient self‐reporting using an electronic diary with a numeric rating scale from 0 (no pain) to 10 (worst possible pain). ^3^Pain NRS was categorised into moderate (4–6) and severe (7–10).
**Table S2.** Mediation analysis of linzagolix on pain reduction from baseline at 24 weeks with menstrual blood loss reductions (continuous) and fibroid volume as mediators (*N* = 1012). ABT, add‐back therapy; CI, confidence interval; FV, fibroid volume; HMB, heavy menstrual bleeding. ^1^For the purposes of the current study, clinically significant pain reduction was defined as decline by ≥ 2 pain categories derived from self‐reported pain on 0–10 scale (none = 0, mild = 1–3, moderate =4–6, severe = 7–10). ^2^Effects are additive in the log odds ratio scale (i.e., mediated effects + residual effects add up to total effect). ^3^Interpretation of odds ratios (OR) > 1: the odds of experiencing a clinically significant reduction in pain are *x* higher (*x* = OR value) for patients in the treatment arm of interest versus patients in the placebo arm. ^4^Percentages of total effect are computed on the log odds ratio scale using the effect sizes estimates. ^5^Residual treatment effects are a combination of the direct treatment effect on pain reduction and the treatment effect mediated by factors other than FV and HMB reductions. *Significant at the 0.0125 level.
**Table S3.** Mediation analysis of linzagolix on pain reduction from baseline at 24 weeks with heavy menstrual bleeding and fibroid volume reductions as mediators among participants with only moderate or severe pain at baseline (*N* = 761). ABT, add‐back therapy; CI, confidence interval; FV, fibroid volume; HMB, heavy menstrual bleeding. ^1^For the purposes of the current study, clinically significant pain reduction was defined as decline by ≥ 2 pain categories derived from self‐reported pain on 0–10 scale (none = 0, mild = 1–3, moderate =4–6, severe = 7–10). ^2^Effects are additive in the log odds ratio scale (i.e., mediated effects + residual effects add up to total effect). ^3^Interpretation of odds ratios (OR) > 1: the odds of experiencing a clinically significant reduction in pain are *x* higher (*x* = OR value) for patients in the treatment arm of interest vs. patients in the placebo arm. ^4^Percentages of total effect are computed on the log odds ratio scale using the effect sizes estimates. ^5^Residual treatment effects are a combination of the direct treatment effect on pain reduction and the treatment effect mediated by factors other than FV and HMB reductions. *Significant at the 0.0125 level.
**Figure S1.** Proportion of participants with reduction in heavy menstrual bleeding at 24 weeks (*N* = 1012). ABT, add‐back therapy.
**Figure S2.** Fibroid volume change from baseline to Weeks 12 and 24 (*N* = 1012). ABT, add‐back therapy. The reduction from baseline is calculated as 100% minus the ratio of current volume (Week 12 and 24) to baseline volume and can be interpreted as percent reduction from baseline.
**Figure S3.** Proportion of participants with reduction in pain at 24 weeks (*N* = 1012). ABT, add‐back therapy.
**Figure S4.** Proportion of participants with only moderate or severe pain at baseline (*N* = 761) who experienced reduction in pain at 24 weeks. ABT, add‐back therapy. Pain numeric rating scale (NRS) was assessed by patient self‐reporting using an electronic diary with a numeric rating scale from 0 (no pain) to 10 (worst possible pain). Pain NRS was categorised into none (0), mild (1–3), moderate (4–6) and severe (7–10).

## Data Availability

The data that support the findings of this study are available from the corresponding author upon reasonable request.
