# Supplementary material for: Pain Reduction in Linzagolix‐Treated Patients With Uterine Fibroids: A Secondary Mediation Analysis of the PRIMROSE 1 and 2 Phase 3 Trials
Source: BJOG. 2025 May 6;132(9):1297–306. doi: 10.1111/1471-0528.18190 (PMC12232536; doi:10.1111/1471-0528.18190)

Supplementary Table 1

	Placebo (N=151)	Linzagolix 100mg (N=148)	Linzagolix 100mg + ABT (N=162)	Linzagolix 200mg (N=157)	Linzagolix 200mg + ABT (N=143)	Total (N=761)
Study ID, n (%)						
PRIMROSE 1	83 (55%)	81 (55%)	91 (56%)	87 (55%)	85 (59%)	427 (56%)
PRIMROSE 2	68 (45%)	67 (45%)	71 (44%)	70 (45%)	58 (41%)	334 (44%)
Age, mean (SD)	42.7 (5.5)	42.5 (5.7)	42.0 (5.8)	41.7 (6.1)	42.2 (5.5)	42.2 (5.7)
Race, n (%)						
Black	56 (37%)	52 (35%)	63 (39%)	59 (38%)	56 (39%)	286 (38%)
White	94 (62%)	90 (61%)	94 (58%)	96 (61%)	84 (59%)	458 (60%)
BMI (kg/m^2), mean (SD)	29.48 (6.64)	30.51 (7.31)	30.33 (6.88)	29.97 (6.80)	30.72 (7.59)	30.19 (7.03)
Total fibroid volume (mL)[1], median (Q1 – Q3)	51 (22 - 133)	68 (30 - 149)	49 (21 - 152)	43 (20 - 114)	50 (20 - 109)	51 (22 - 129)
Pain NRS[2], mean (SD)	6.62 (1.90)	6.81 (1.80)	6.78 (1.93)	7.18 (1.84)	6.92 (1.77)	6.86 (1.86)
Pain[3], n (%)						
moderate	74 (49%)	63 (43%)	73 (45%)	59 (38%)	58 (41%)	327 (43%)
severe	77 (51%)	85 (57%)	89 (55%)	98 (62%)	85 (59%)	434 (57%)
Menstrual blood loss (mL), median (Q1 – Q3)	167 (124 - 239)	171 (125 - 272)	160 (120 - 260)	167 (127 - 274)	164 (118 - 232)	165 (123 - 257)
Baseline use of analgesic medications, n (%)	18 (12%)	25 (17%)	24 (15%)	21 (13%)	21 (15%)	109 (14%)

Supplementary Table 2

Treatment effect on clinically significant pain reduction[1]	Odds Ratio (95% CI)[2,3]	P-values	Percentage of total effects[4]
**Linzagolix 100mg vs placebo**			
Total effect of linzagolix 100mg vs placebo	4.03 (1.76 - 9.67)	0.002*	
Effect mediated by FV and MBL reductions	1.77 (1.25 - 3.04)	0.013	41%
Effect mediated by FV reduction	1.09 (1.00 - 1.20)	0.042	6%
Effect mediated by MBL reduction	1.64 (1.18 - 2.80)	0.029	35%
Residual treatment effect[5]	2.27 (1.09 - 5.36)	0.034	59%
**Linzagolix 100mg + ABT vs placebo**			
Total effect of linzagolix 100mg + ABT vs placebo	5.68 (2.53 - 14.76)	<0.001*	
Effect mediated by FV and MBL reductions	2.57 (1.59 - 5.47)	0.003*	54%
Effect mediated by FV reduction	1.03 (0.95 - 1.11)	0.520	1%
Effect mediated by MBL reduction	2.49 (1.55 - 5.25)	0.004*	53%
Residual treatment effect[5]	2.21 (1.08 - 5.19)	0.038	46%
**Linzagolix 200mg vs placebo**			
Total effect of linzagolix 200mg vs placebo	9.64 (4.18 - 25.26)	<0.001*	
Effect mediated by FV and MBL reductions	3.12 (1.88 - 6.88)	0.001*	50%
Effect mediated by FV reduction	1.20 (1.05 - 1.40)	0.002*	8%
Effect mediated by MBL reduction	2.60 (1.59 - 5.74)	0.005*	42%
Residual treatment effect[5]	3.09 (1.54 - 7.02)	0.004*	50%
**Linzagolix 200mg + ABT vs placebo**			
Total effect of linzagolix 200mg + ABT vs placebo	6.32 (2.74 – 17.32)	<0.001*	
Effect mediated by FV and MBL reductions	3.13 (1.80 - 7.34)	0.002*	62%
Effect mediated by FV reduction	1.08 (1.00 - 1.20)	0.066	4%
Effect mediated by MBL reduction	2.89 (1.69 - 6.62)	0.003*	58%
Residual treatment effect[5]	2.02 (1.00 - 4.51)	0.050	38%

Supplementary Table 3

Treatment effect on clinically significant pain reduction[1]	Odds Ratio (95% CI)[2,3]	P-values	Percentage of total effects[4]
**Linzagolix 100mg vs placebo**			
Total effect of linzagolix 100mg vs placebo	3.61 (1.62 - 8.68)	0.003*	
Effect mediated by FV and HMB reductions	1.63 (1.24 - 2.21)	0.001*	38%
Effect mediated by FV reduction	1.10 (0.99 - 1.25)	0.088	7%
Effect mediated by HMB reduction	1.48 (1.19 - 1.94)	0.002*	31%
Residual treatment effect[5]	2.22 (1.02 - 5.07)	0.046	62%
**Linzagolix 100mg + ABT vs placebo**			
Total effect of linzagolix 100mg + ABT vs placebo	5.40 (2.54 - 12.98)	<0.001*	
Effect mediated by FV and HMB reductions	2.36 (1.68 - 3.64)	<0.001*	51%
Effect mediated by FV reduction	1.06 (0.96 - 1.21)	0.230	4%
Effect mediated by HMB reduction	2.22 (1.58 - 3.34)	<0.001*	47%
Residual treatment effect[5]	2.29 (1.02 - 5.42)	0.044	49%
**Linzagolix 200mg vs placebo**			
Total effect of linzagolix 200mg vs placebo	10.69 (4.81 - 27.38)	<0.001*	
Effect mediated by FV and HMB reductions	2.82 (1.95 - 4.65)	<0.001*	44%
Effect mediated by FV reduction	1.28 (1.09 - 1.59)	0.002*	10%
Effect mediated by HMB reduction	2.21 (1.61 - 3.35)	<0.001*	34%
Residual treatment effect[5]	3.78 (1.68 - 9.29)	0.002*	56%
**Linzagolix 200mg + ABT vs placebo**			
Total effect of linzagolix 200mg + ABT vs placebo	6.82 (3.39 - 17.89)	<0.001*	
Effect mediated by FV and HMB reductions	2.76 (1.89 - 4.51)	<0.001*	53%
Effect mediated by FV reduction	1.08 (0.96 - 1.26)	0.178	4%
Effect mediated by HMB reduction	2.56 (1.78 - 4.13)	<0.001*	49%
Residual treatment effect[5]	2.47 (1.14 - 5.81)	0.018	47%

Supplementary Figure 1

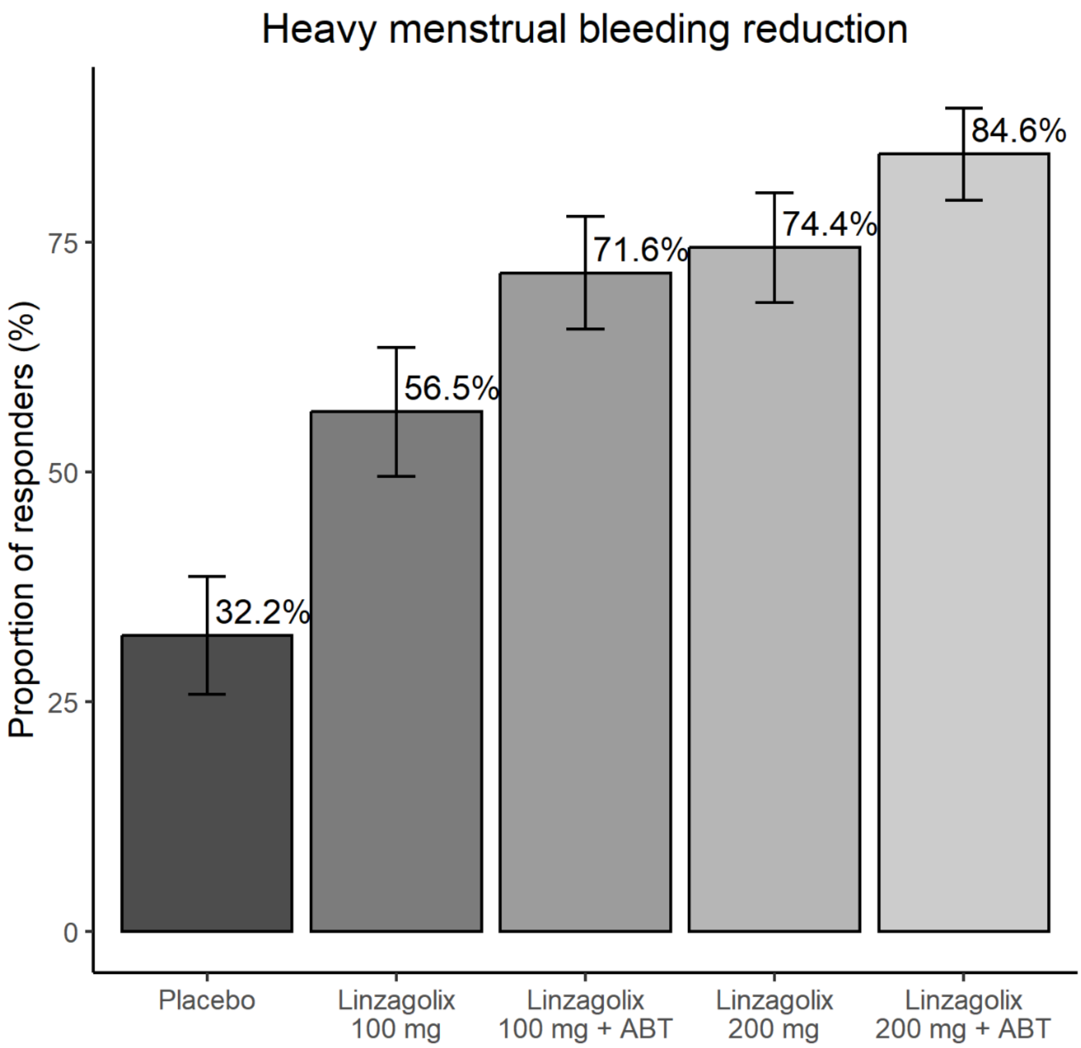

Supplementary Figure 2

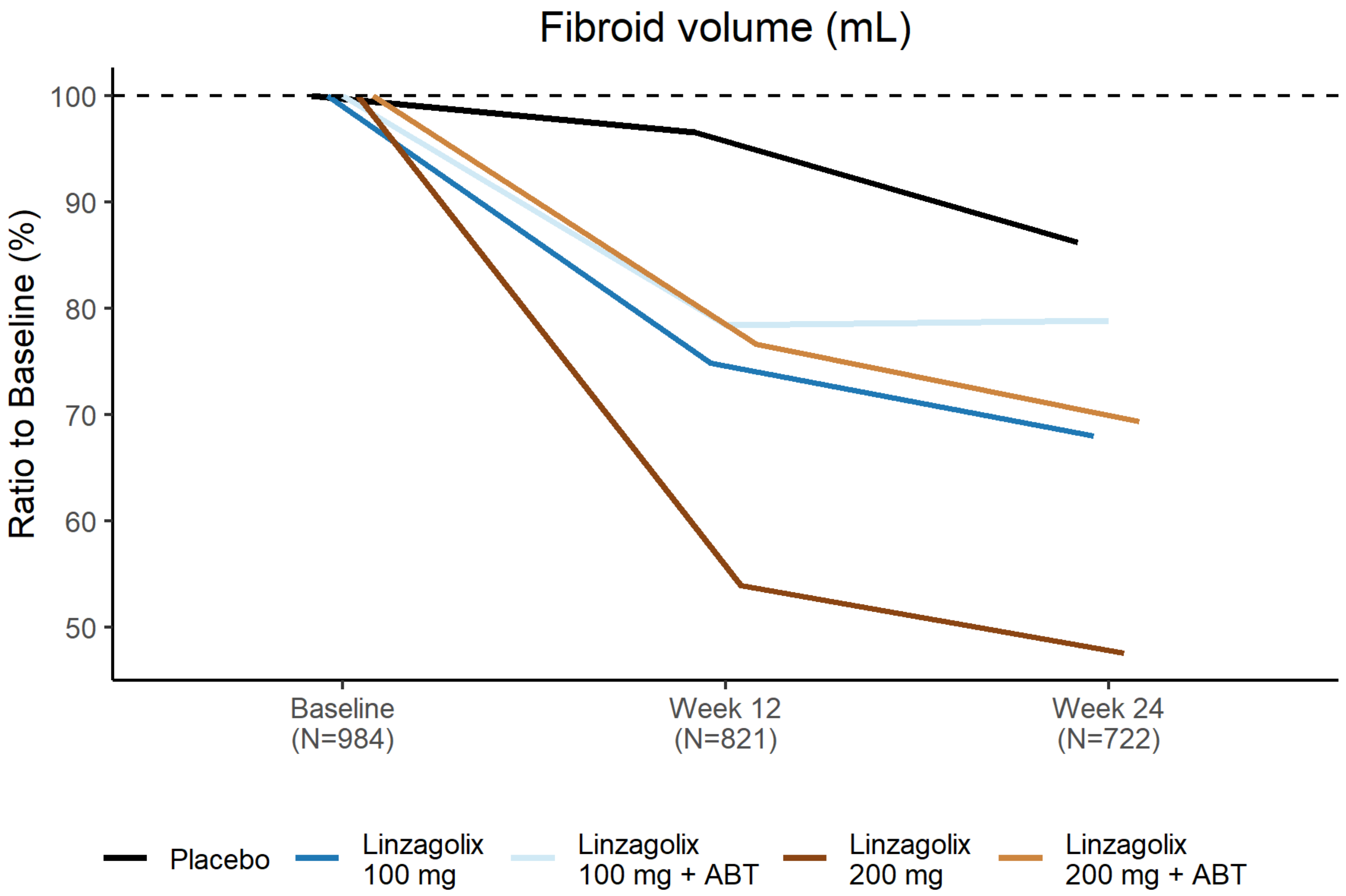

Supplementary Figure 3

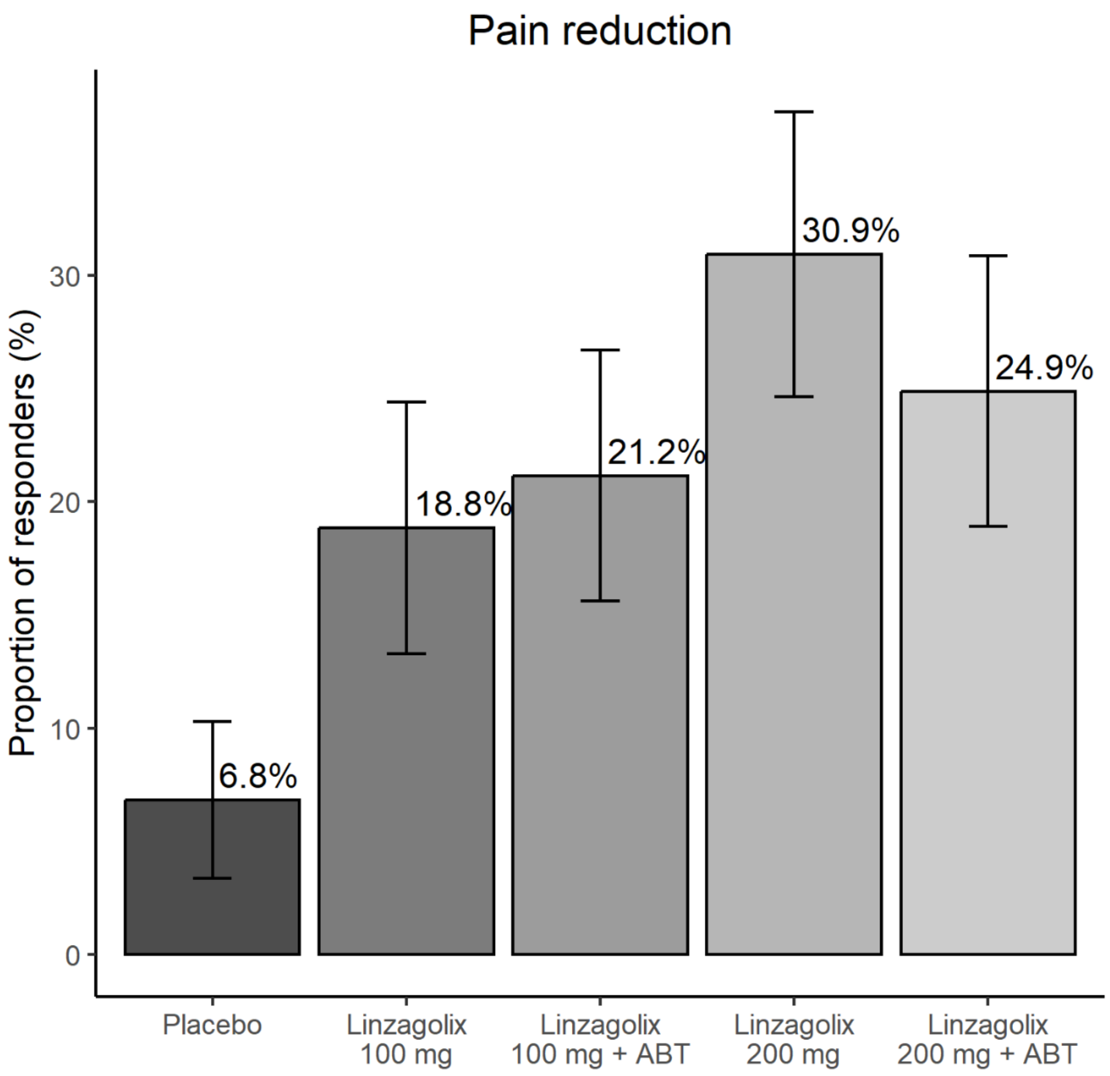

Supplemental Figure 4

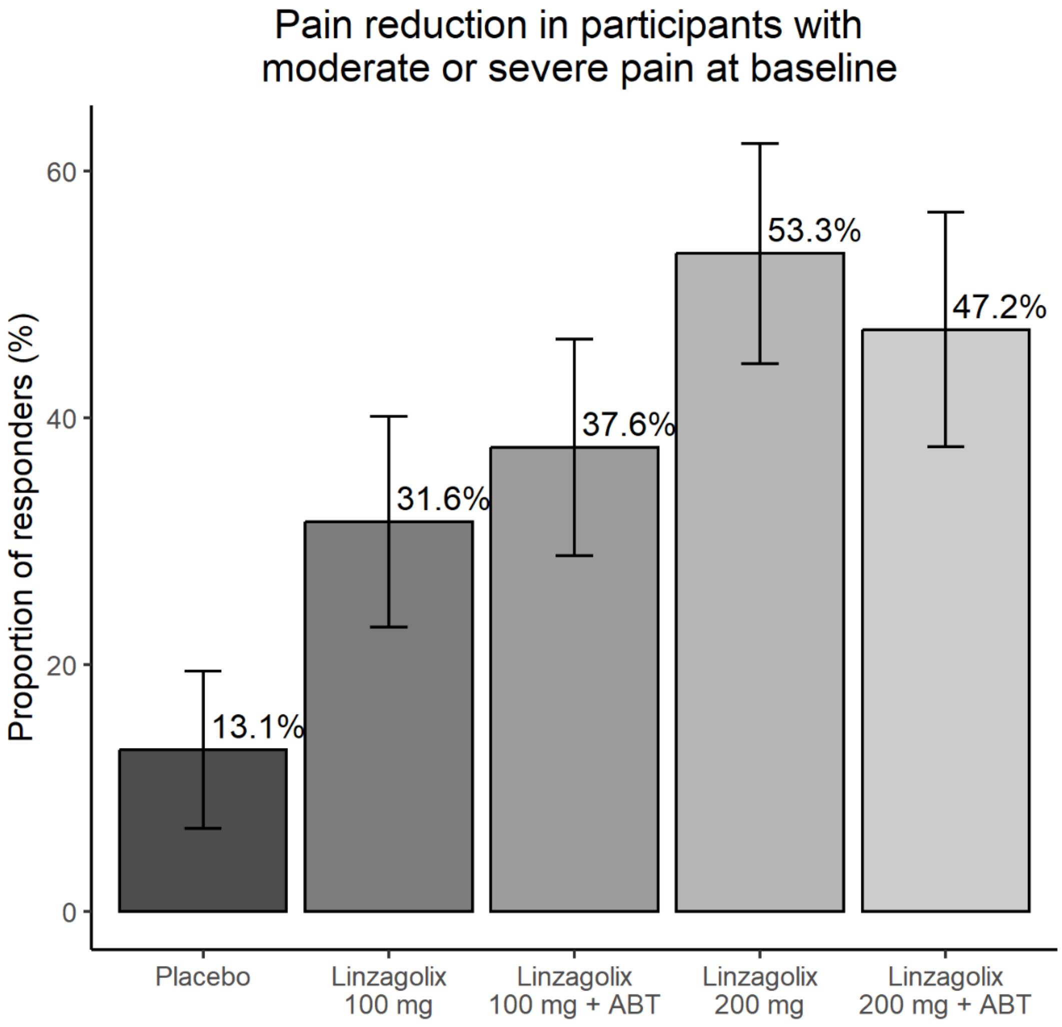

Supplement: Supplementary file 1 — Appendix S1. Table S1. Demographic and clinical characteristics of participants with only moderate or severe pain at baseline (N = 761). ABT, add‐back therapy; BMI, body mass index; NRS, numeric rating scale; SD, standard deviation 1Fibroid dimensions were estimated using ultrasonography; volumes were calculated by the prolate ellipsoid formula: length×height×width×0.523. Up to the three largest fibroids were included in the total FV calculation. 1 mL = 1 cm3. 2Pain numeric rating scale (NRS) was assessed by patient self‐reporting using an electronic diary with a numeric rating scale from 0 (no pain) to 10 (worst possible pain). 3Pain NRS was categorised into moderate (4–6) and severe (7–10). Table S2. Mediation analysis of linzagolix on pain reduction from baseline at 24 weeks with menstrual blood loss reductions (continuous) and fibroid volume as mediators (N = 1012). ABT, add‐back therapy; CI, confidence interval; FV, fibroid volume; HMB, heavy menstrual bleeding. 1For the purposes of the current study, clinically significant pain reduction was defined as decline by ≥ 2 pain categories derived from self‐reported pain on 0–10 scale (none = 0, mild = 1–3, moderate =4–6, severe = 7–10). 2Effects are additive in the log odds ratio scale (i.e., mediated effects + residual effects add up to total effect). 3Interpretation of odds ratios (OR) > 1: the odds of experiencing a clinically significant reduction in pain are x higher (x = OR value) for patients in the treatment arm of interest versus patients in the placebo arm. 4Percentages of total effect are computed on the log odds ratio scale using the effect sizes estimates. 5Residual treatment effects are a combination of the direct treatment effect on pain reduction and the treatment effect mediated by factors other than FV and HMB reductions. *Significant at the 0.0125 level. Table S3. Mediation analysis of linzagolix on pain reduction from baseline at 24 weeks with heavy menstrual bleeding and fibroid volume reduction [file BJO-132-1297-s001.pdf]
